# Detection of 40 bp insertion-deletion (INDEL) in mitochondrial control region among sambar (*Rusa unicolor*) populations in India

**DOI:** 10.1186/s13104-015-1573-2

**Published:** 2015-10-19

**Authors:** Sandeep Kumar Gupta, Ajit Kumar, Ajay Gaur, Syed Ainul Hussain

**Affiliations:** Wildlife Institute of India, Chandrabani, Dehra Dun, 248 001 India; Laboratory for the Conservation of Endangered Species (LaCONES), CSIR-Centre for Cellular and Molecular Biology, Hyderabad, India

**Keywords:** Sambar, Genetic variation, mtDNA control region, Insertion-deletion

## Abstract

**Background:**

The mitochondrial DNA (mtDNA) control region is extensively used in the phylogeography of species. We examined sequence variations in the mtDNA control region of sambar (*Rusa unicolor*) populations from the South, Central and North India.

**Results:**

Most of the samples collected from the south India exhibited a 40 bp insertion in the mtDNA control region. This insertion was not observed in the North and Central Indian populations.

**Conclusion:**

This study provided a potential marker for molecular screening and identification of sambar populations in the form of a distinct 40 bp insertion. Some populations in South India did not exhibit this insertion. It indicates that there could be an ecological barrier that might be preventing the expansion of insertion-positive sambar population.

## Background

The sambar (*Rusa unicolor*) is the largest cervid species in Southeast Asia. In India, it is widely distributed from the Himalayan foothills to the southern limits. Seven subspecies of sambar are recognised, with *R. u. unicolor* occurring in India and Sri Lanka [1]. Despite a large distribution range, very limited information is available on genetic variations for this species.

The mitochondrial DNA (mtDNA) control region has been used extensively in studying the population genetics of wild species, for example the tiger, *Panthera tigris* [2]; wild pig, *Sus scrofa* [3]; cervids [4]; sika deer, *Cervus nippon* [5]; roe deer, *Capreolus capreolus* [6]; white-tailed deer, *Odocoileus virginianus* [7]; Chinese water deer, *Hydropotes inermis inermis* [8] and black muntjac, *Muntiacus crinifrons* [9]. In the present study, we investigated genetic variations among sambar populations of selected zones of India. Sequences of the mtDNA hypervariable region *I* (HVR-*I*) were compared among the sambar populations of the South, Central and North India for evaluation of genetic variation.

## Results

**Fig. 1:**
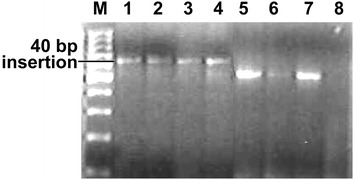
Gel image showing 40 bp longer PCR amplification from the samples of South India (lanes *1*–*4*) and shorter amplification from the samples of Central and North India (lanes *5*–*7*). Lane *M* is 100 bp DNA ladder

**Fig. 2:**
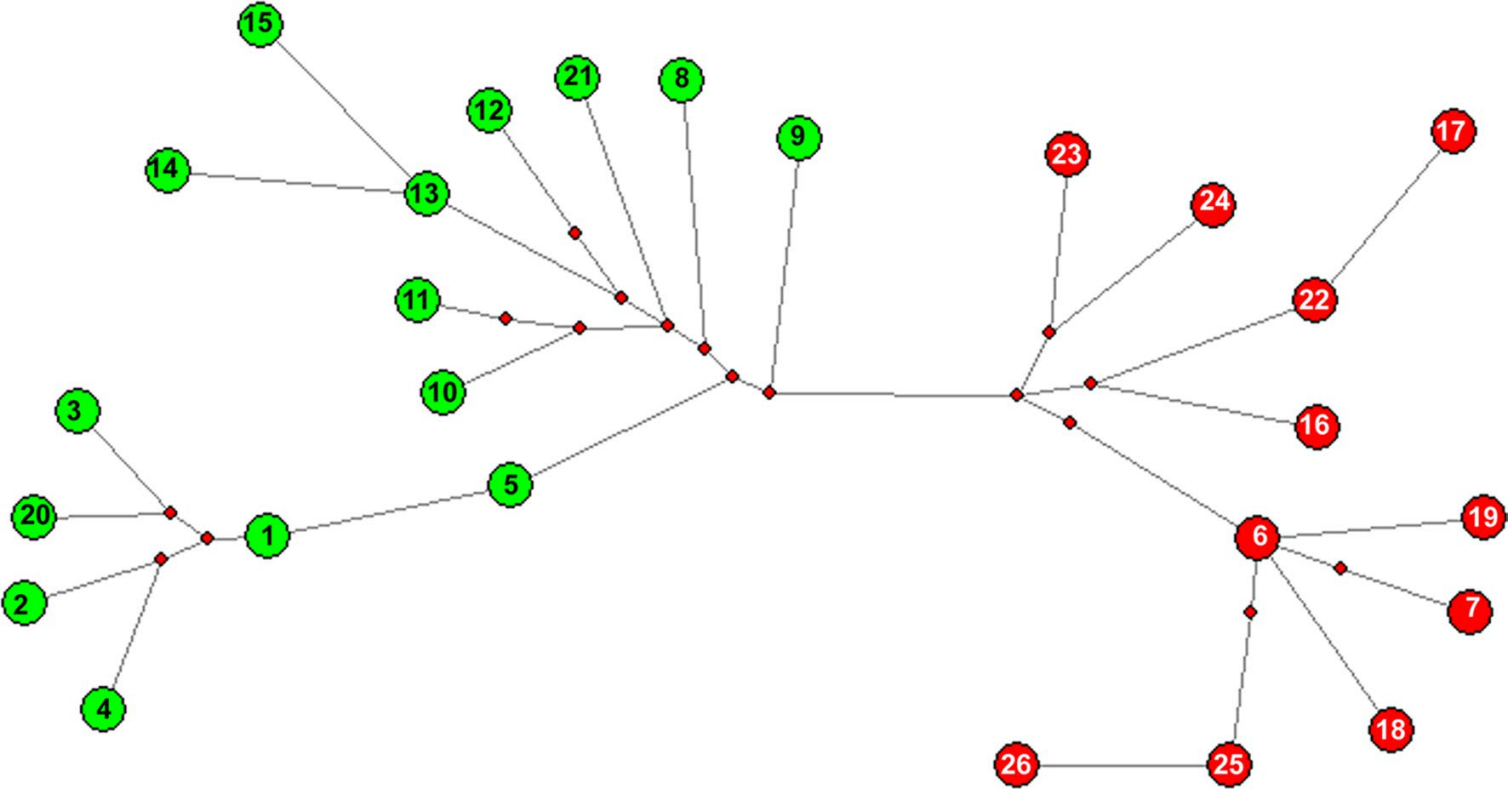
Median-joining (MJ) network tree demonstrating distinct clustering of 40 bp insertion positive (*green*) and negative (r*ed*) haplotypes. Haplotype numbers are given in the *circle* and described in Table 1

Overall, the mtDNA HVR-*I* region demonstrated high variability among sambar populations, and 26 distinct haplotypes were identified (Table 1). PCR amplifications that were 40 bp longer than the expected size were obtained with DNA extracted from 23 samples from South India (Fig. 1). Three samples from South India and all 38 samples from Central and North India yielded amplifications of the expected size. It indicated that the majority of the animals of the South Indian population have a unique feature in the mtDNA control region. We obtained 543–623 bp sequences of the control region using a cervid specific primer pair. After alignment of the sequences, 23 samples of South India exhibited a unique 40 bp insertion after nucleotide (nt) position 233 (Table 1). This insertion in a majority of the South Indian samples indicated that there is a significant genetic variation in this population. Four out of the 23 insertion-positive south Indian samples were collected from a confined population from a zoological park in Goa. The 40 bp insertion was absent in three out of eight samples collected from Kalakkad-Mundunthurai Tiger Reserve (KMTR), Tamil Nadu. The 40 bp insertion was not observed in the samples of central and north Indian populations. Therefore, the 40 bp deletion in the control region after nt position 233 appears to be a potential marker for genetic screening and identification of Central and North Indian populations. The haplotype (RUC1–RUC26) sequences were submitted to GenBank (accession numbers KF133981-99 and KF648589-95, Table 1). Haplotypes RUC1–RUC5, RUC8–RUC15, RUC20 and RUC21 were molecular signatures of the insertion-positive South Indian population. The most frequent haplotype was RUC17, which was observed in 24 individuals from Central and North India. Haplotypes RUC2 and RUC18 were observed in four individuals from the South and Central India and RUC19 in three individuals from Central India (Table 1). The pairwise distance and percentage similarity matrices showed wide ranges of 0.002–0.113 and 91.60–99.83, respectively (Table 2). We observed 62 polymorphic sites (S) among 26 haplotypes. The average number of nucleotide differences (k) and nucleotide diversity (π) were 24.00615 and 0.04479, respectively. Median-joining (MJ) network tree exhibited a distinct clustering of insertion-positive (green colour) and insertion-negative (red colour) populations (Fig. 2).

**Table 1:**
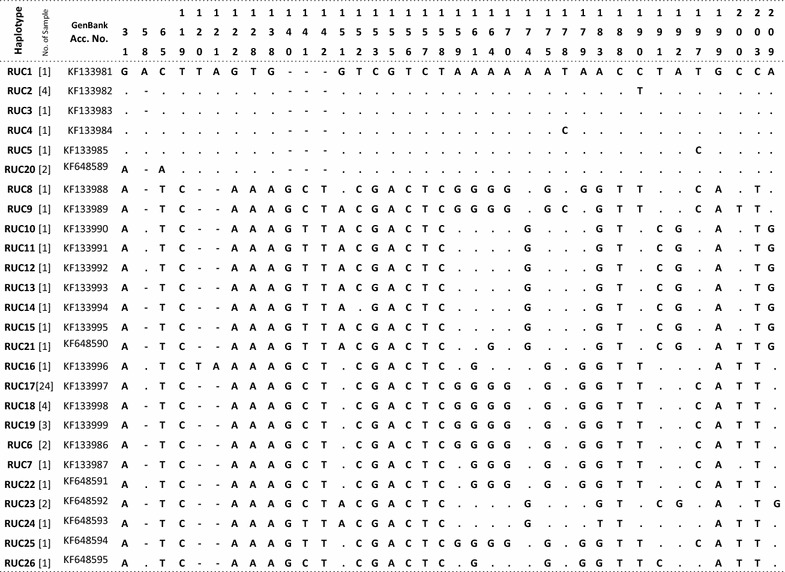

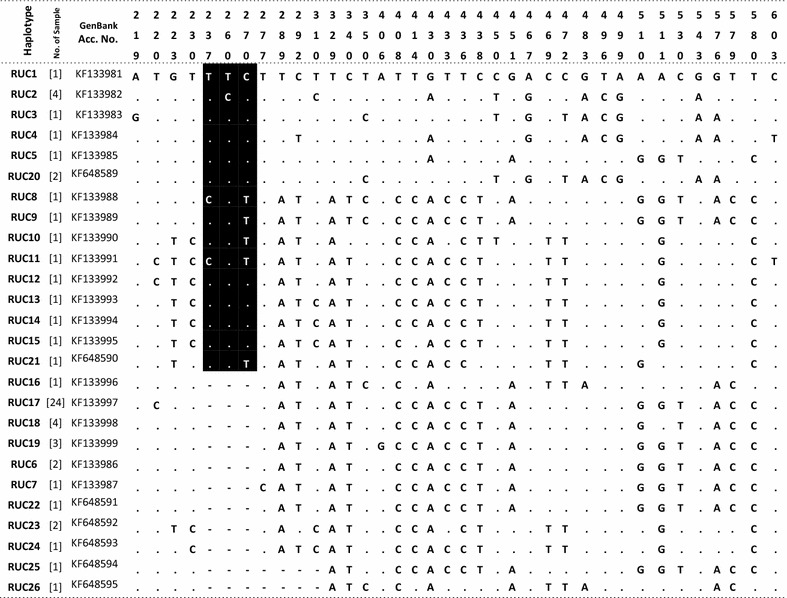
Table showing 26 haplotypes (RUC1-26) with number of repeat in square bracket []

Dot represent similarity with first sequence and hyphen (-) represent gap. Numerics at top represent position of variable nucleotide. Numbering of nucleotide started from the first base of control region. Alphanumeric are the NCBI GenBank accession number of haplotype sequences. Dark shade represents the 40 bp insertion

**Table 2:**
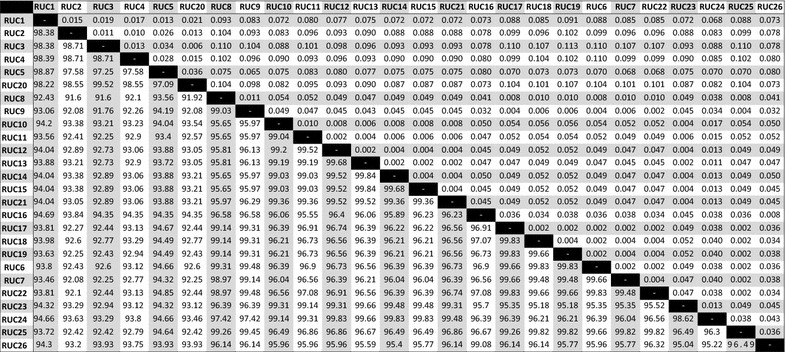
Table showing pair wise distance (above diagonal) and percent similarity (below diagonal) amongst control region sequences generated by deer specific primer Balakrishnan et al. [10]

## Discussion and conclusions

In several studies, insertion–deletion (INDEL) markers have been used in population genetics and forensics [10–12] as it is easy to use. In the present study, we used INDEL to describe a unique 40 bp insertion in the HVR-*I* (control) region that is found mostly in the sambar population from the South India. This unique molecular feature differentiated it from the other populations of sambar from India suggesting further investigation. It is apparent from genetic data that INDEL has a significant role in population structuring in sambar.

This study provided further insight into the genetic makeup of sambar. Analysis of samples collected from various parts of India revealed a high level of genetic variations among different populations. The percentage similarity and pairwise distance indicate that the insertion-positive haplotypes (RUC1–RUC5, RUC8–RUC15, RUC20 and RUC21) were significantly related and had a higher genetic distance from the other haplotypes (Table 2). We suspect that there could be a possible ecological barrier operating, which is separating majority of the South Indian population from the North and Central India. The 40 bp INDEL in the mtDNA region is a rapid marker for genetic screening and identification of these populations using a simple PCR and sequencing-based analysis.

## Methods

### Sampling and DNA extraction

**Fig. 3:**
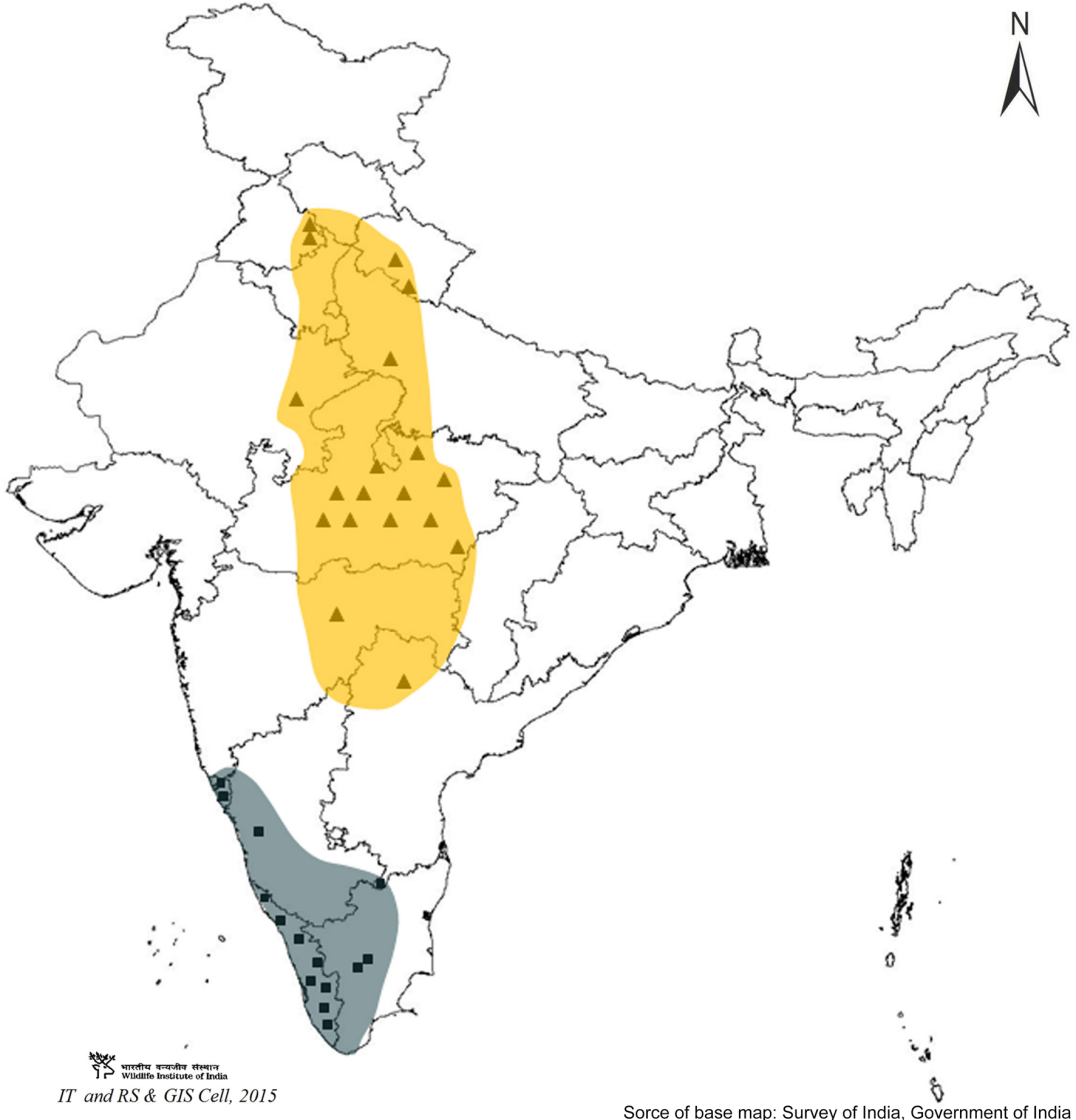
Locations of sampling site are shown with *square* from south India, which has 40 bp insertion, and *triangle* for populations from central and north India those do not have this insertion. *Gray* and *yellow sheds* represent tentative sites for insertion positive and negative populations, respectively. Source of the base map of India was Survey of India, Government of India and it was procured by Wildlife Institute of India for academic research and illustration purpose

A total of 64 biological samples (54 tissue samples and ten fecal samples) were used in this study. Of these, 26 were from South India, and 38 were from Central and North India (Fig. 3). Out of the 64 samples, 36 had been seized from poachers and forwarded by enforcement agencies to us for forensic validation. The confiscated biological samples were validated for species through sequence analysis of the mtDNA cytochrome *b* gene [13]. Biological samples were also collected from national parks and captive populations of sambar after obtaining permission from the Principal Chief Conservation of Forests (Wildlife) and Chief Wildlife Warden of the States of Uttarakhand, Madhya Pradesh, Andhra Pradesh, Kerala, Tamil Nadu, Karnataka and Goa. International regulations such as the Convention on the Trade in Endangered Species of Wild Fauna and Flora (CITES, http://www.cites.org) and Convention on Biological Diversity (CBD, http://www.cbd.int/convention) were also complied with during the study. High standards were maintained during sample collection, and no animals were harmed during this study. Six tissue samples and one shed antler sample were collected from predator kills from Panna Tiger Reserve, Madhya Pradesh. Seven and four individual’s hair samples were collected from confined populations in deer parks in Andhra Pradesh and Goa, respectively. These hair samples were collected from the metal grills and concrete wall of the captive area. Ten faecal samples were collected from forests in Kerala and Tamil Nadu. Since no animal handling was involved, approval of animal ethical committees was not required. Whole genomic DNA was extracted using phenol–chloroform [14]. A standardized protocol developed for extraction of DNA from non-invasive sampling was followed [15].

### PCR amplification

The DNA extracted from above samples was used for PCR amplification of an approximately 600 bp long sequence of the mtDNA control region. A primer pair specific to the family Cervidae (deer) was used [10]. The sequences of the primer are CervtPro “CCACYATCAACACCCAAAGC” and CervCRH “GCCCTGAARAAAGAACCAGATG”. PCR reactions were carried out in 20 µl reaction volumes using 1 × PCR buffer (10 mM Tris–HCl, pH 8.3, and 50 mM KCl), 1.5 mM MgCl_2_, 1 × BSA, 100 µM dNTPs, 4 pmol of each primer, 0.5 U AmpliTaq Gold DNA polymerase enzyme (Invitrogen Inc.) and 1 µl (~30 ng) of template DNA. The PCR conditions were initial denaturation at 95 °C for 10 min, followed by 35 cycles of denaturation at 95 °C for 45 sec, annealing at 54 °C for 40 sec and extension at 72 °C for 75 sec. The final extension was at 72 °C for 10 min. The efficiency and reliability of the PCR reactions were monitored using control reactions. PCR amplification was confirmed by electrophoresis on 2.2 % agarose gel stained with ethidium bromide (0.5 mg/ml) and visualised under a UV transilluminator.

### DNA sequencing and analysis

The PCR products were treated with exonuclease-*I* and shrimp alkaline phosphatase for 15 min each at 37 and 80 °C. A BigDye terminator kit (version3.1) and an ABI 3130 Genetic Analyzer (Applied Biosystems) were used to generate DNA sequences from both the directions. The generated sequences were aligned by eye using Clustal W [16], available in the BioEdit package [17]. Since the primer pair used in this study amplifies approximately 40–50 bases of Proline tRNA at the beginning of the PCR reaction, the initial sequence of Proline tRNA was deleted from the aligned sequences. Hence, the start sequence examined in this study was the first nucleotide of the control region. The aligned sequences of the control region were subjected to haplotype analysis using DnaSP [18] (Table 1). A percentage similarity matrix and pairwise distance matrix were generated using Clustal W [16] and MEGA 5 [19] (Table 2). Network 4.613 software (http://www.fluxus-engineering.com) was used to generate median-joining network tree (Fig. 3).

## Abbreviations

CBD: Convention on Biological Diversity
CITES: Convention on the Trade in Endangered Species of Wild Fauna and Flora
HVR-*I*: hypervariable region *I*
INDEL: insertion–deletion
KMTR: Kalakkad-Mundunthurai Tiger Reserve
MJ: median-joining
mtDNA: mitochondrial DNA
nt: nucleotide
